# Expanded complement of Niemann-Pick type C2-like protein genes in *Clonorchis sinensis* suggests functions beyond sterol binding and transport

**DOI:** 10.1186/s13071-020-3910-0

**Published:** 2020-01-23

**Authors:** Marziyeh Anari, Andreas J. Stroehlein, Ross S. Hall, Bill C. H. Chang, Robin B. Gasser, Neil D. Young

**Affiliations:** Department of Veterinary Biosciences, Melbourne Veterinary School, Faculty of Veterinary and Agricultural Sciences, The University of Melbourne, Parkville, VIC 3010 Australia

**Keywords:** *Clonorchis sinensis*, Comparative genomics, Niemann-pick type C2, NPC2, Functional protein annotation, Adaptation

## Abstract

**Background:**

The parasitic flatworm *Clonorchis sinensis* inhabits the biliary tree of humans and other piscivorous mammals. This parasite can survive and thrive in the bile duct, despite exposure to bile constituents and host immune attack. Although the precise biological mechanisms underlying this adaptation are unknown, previous work indicated that Niemann-pick type C2 (NPC2)-like sterol-binding proteins might be integral in the host-parasite interplay. Expansions of this family in some invertebrates, such as arthropods, have shown functional diversification, including novel forms of chemoreception. Thus, here we curated the NPC2-like protein gene complement in *C. sinensis*, and predicted their conserved and/or divergent functional roles.

**Methods:**

We used an established comparative genomic-bioinformatic approach to curate NPC2-like proteins encoded in published genomes of Korean and Chinese isolates of *C. sinensis*. Protein sequence and structural homology, presence of conserved domains and phylogeny were used to group and functionally classify NPC2-like proteins. Furthermore, transcription levels of NPC2-like protein-encoding genes were explored in different developmental stages and tissues.

**Results:**

Totals of 35 and 32 *C. sinensis* NPC2-like proteins were predicted to be encoded in the genomes of the Korean and Chinese isolates, respectively. Overall, these proteins had low sequence homology and high variability of sequence alignment coverage when compared with curated NPC2s. Most *C. sinensis* proteins were predicted to retain a conserved ML domain and a conserved fold conformation, with a large cavity within the protein. Only one protein sequence retained the conserved amino acid residues required in bovine NPC2 to bind cholesterol. Non-canonical *C. sinensis* NPC2-like protein-coding domains clustered into four distinct phylogenetic groups with members of a group frequently encoded on the same genome scaffolds. Interestingly, NPC2-like protein-encoding genes were predicted to be variably transcribed in different developmental stages and adult tissues, with most being transcribed in the metacercarial stage.

**Conclusions:**

The results of the present investigation confirms an expansion of NPC2-like proteins in *C. sinensis*, suggesting a diverse array of functions beyond sterol binding and transport. Functional explorations of this protein family should elucidate the mechanisms enabling the establishment and survival of *C. sinensis* and related flukes in the biliary systems of mammalian hosts.
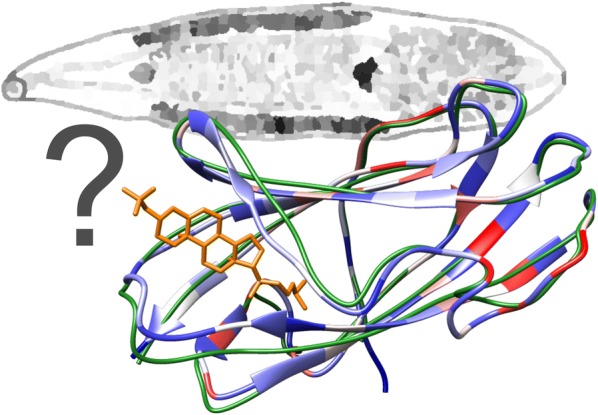

## Background

Parasitic flatworms (phylum Platyhelminthes; class Trematoda) are responsible for neglected tropical diseases (NTDs) that affect more than 750 million humans and other mammals throughout the world, particularly in Southeast Asia and the Western Pacific regions [1–3]. Important trematode species include *Clonorchis sinensis* and *Opisthorchis* spp. [3, 4]. The consumption of fish containing the infective developmental stage (metacercaria) of these liver flukes leads to an infection that, if chronic, can cause serious hepatobiliary diseases in humans, including clonorchiasis (*C. sinensis*) or opisthorchiasis (*Opisthorchis* spp.), particularly in Asia [3, 5–7]. For example, *C. sinensis* impacts more than 35 million people across China, Japan, Korea and Vietnam [8, 9], and has been classified as a Class I carcinogen by the International Agency for Research on Cancer (IARC) [10]. Despite the importance of this parasite, the molecular mechanisms that govern or modulate the interactions between *C. sinensis* and its host animals remain largely unknown.

When piscivorous mammals (e.g. humans, dogs and cats) consume fish infected by *C. sinensis*, metacercariae excyst in the duodenum, and juveniles migrate and develop to adult flukes in the biliary system [11, 12]. The adult flukes can alter biliary duct pathology *via* mechanical irritation and by releasing molecules to facilitate feeding [13]. Furthermore, as the adult flukes grow and migrate, they can obstruct the bile duct and elevate bile duct pressure [6]. Clonorchiasis often leads to chronic hepatobiliary illness and can induce cholangiocarcinoma (CCA), a malignant cancer of the biliary system [1, 5, 14]. To better understand the pathogenesis of clonorchiasis and CCA, and to assist in efforts to control the parasite causing these diseases, researchers have explored the function of *C. sinensis* proteins, including proteins likely to be important for establishment and survival in the human biliary system.

In bile, cholesterol and phospholipids are abundant, and lipid-binding proteins (LBPs) have been proposed to play an important role in maintaining the chemical homeostasis of liver flukes in the bile duct [15]. Interestingly, gene duplication events have led to more than 20 copies of genes encoding homologues of a lipid-binding protein, Niemann-Pick type C2 (NPC2), in the genomes of *C. sinensis* and *O. viverrini* [16–18]. In most eukaryotes, NPC2 is encoded by a single copy gene. In some mammals, this protein binds cholesterol and other lipids and transports them out of the lysosome to other parts of the cell [19]. In arthropods, duplication and genetic differentiation of NPC2 genes [20–23] have led to new molecular functions, including chemoreception *via* binding to semiochemical and other volatile compounds [23–25]. Related proteins that contain a conserved myeloid differentiation factor-2 (MD-2)-related lipid-binding (ML) domain are also reported to play diverse roles in lipid metabolism, innate immunity and/or chemoreception in arthropods [20, 26]. Despite the substantial expansion of the NPC2-like protein family in liver flukes, almost nothing is known, at the molecular level, about their biological functions in such parasites and/or the regulation of host-pathogen interactions. Here, we employed a bioinformatic workflow to predict, curate and annotate NPC2-like proteins encoded in the draft genomes of a Korean and a Chinese isolate of *C. sinensis*.

## Methods

### Inference of NPC2-like protein sequences

**Fig. 1:**
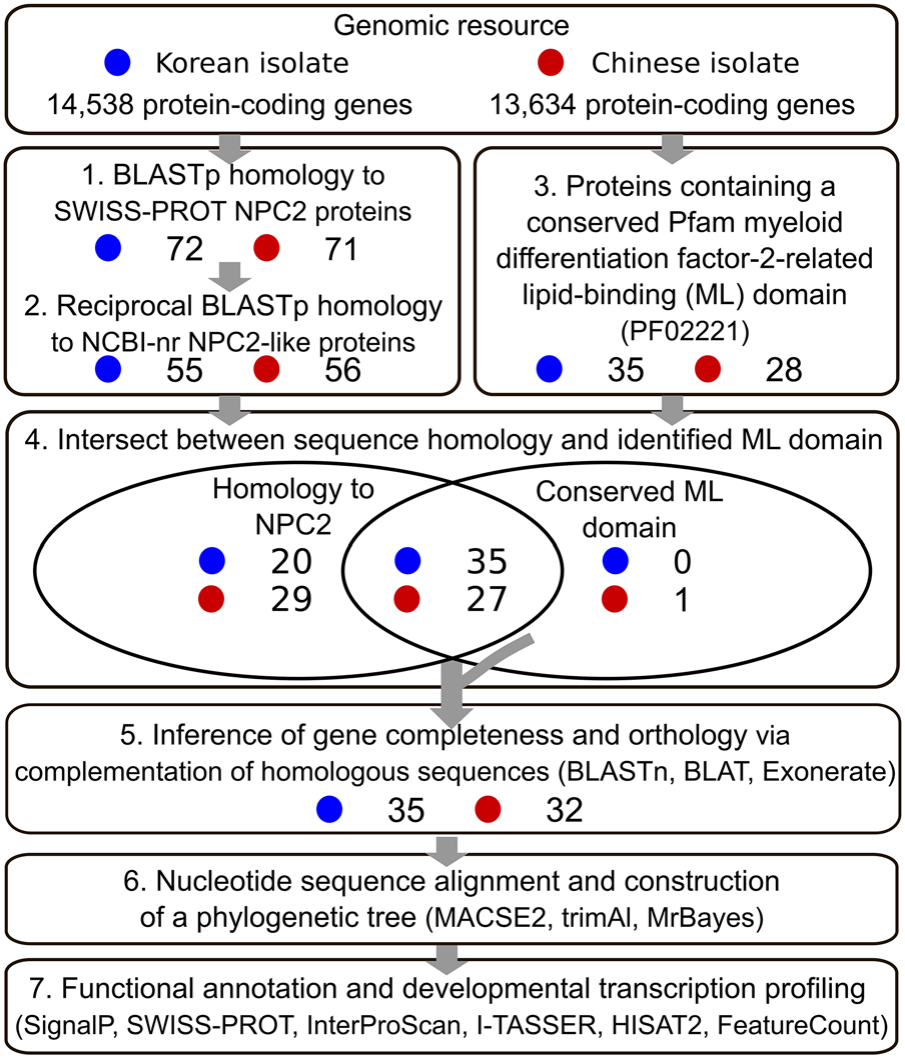
Summary of workflow and results for the prediction, curation, and annotation of NPC2-like proteins in the genomes of a Korean and Chinese isolate of *Clonorchis sinensis*. Initially, putative *C. sinensis* NPC2-like proteins were identified using BLASTp sequence homology searches against curated NPC2 proteins submitted to the SWISS-PROT database (step 1). Selected proteins with a reciprocal best match to NPC2-like proteins submitted to NCBI-nr database were then identified (step 2). Next, *C. sinensis* predicted proteins with a conserved myeloid differentiation factor-2 (MD-2)-related lipid-binding (ML) domain (PF02221) were identified using InterProScan (step 3). The intersect between NPC2 protein sequence homology and retention of a conserved ML domain was then used to select putative NPC2-like proteins. Reciprocal BLASTn sequence homology across gene regions was used to infer orthology and incomplete homologous sequences were complemented in both isolates of *C. sinensis* using BLAT and Exonerate (step 5). Phylogenetic relationships among identified *C. sinensis* NPC2-like protein-encoding genes was then determined by employing MACSE2, TrimAl and MrBayes (step 6). Last, all NPC2-like proteins identified were functionally annotated using SignalP-5.0, SWISS-PROT and InterProScan databases, as well as I-TASSER, to predict tertiary structures. Transcription in different development stages and adult tissues was inferred using HISAT2 and FeatureCounts (step 7). The number of Korean (blue) and Chinese (red) *C. sinensis* sequences retained in each step are indicated

Two *C. sinensis* genomes, one assembled from a Korean isolate (gene accession numbers beginning with “Cs-k2”; BioProject ID: PRJNA386618) [17] and one from a Chinese isolate (gene accession numbers beginning with “csin”; BioProject ID: PRJNA72781) [18], and their gene annotations were downloaded from the WormBase ParaSite database (v.13; accessed 20 May 2019) [27]. NPC2-like homologues in *C. sinensis* were initially identified (Fig. 1, steps 1-3). First, homologues of nine curated NPC2 proteins available in the SWISS-PROT database (Table 1; accessed 20 May 2019) [28] were identified in the *C. sinensis* proteomes using BLASTp v.2.2.29 (E-value cut-off: 10) [29]. Second, a reciprocal BLASTp (E-value cut-off: 10) search of homologues of NPC2 proteins from SWISS-PROT against the NCBI non-redundant protein database (NCBI-nr; accessed 20 May 2019) [30] was performed. *Clonorchis sinensis* NPC2-like protein homologues matching proteins submitted to NCBI-nr and annotated as “Niemann-pick C2 protein”, “NPC intracellular cholesterol transport 2”, “Epididymal secretory protein E1” or “Epididymal secretory protein E1-like”, or that lacked a protein description (unnamed or hypothetical proteins), were retained. Third, we searched for conserved domains in the predicted *C. sinensis* proteome using the program InterProScan v.5.15.54 [31], utilising the Pfam database v.27.0 [32]. Proteins with a conserved ML domain (Pfam identifier PF02221) were retained. Numbers of *C. sinensis* proteins with NPC2 homologues and/or a conserved ML domain were displayed in a Venn diagram employing the Intervene tool [33]. Heatmaps of BLASTp and InterProScan bit scores were created using the R package *ggtree* v.1.16.6 [34].

**Table 1:** Nine curated Niemann-Pick C2 (NPC2) proteins in the SWISS-PROT database used for identification of *C. sinensis* NPC2-like protein homologues. Protein length, signal peptide position and the most similar solved crystal structure are given for each sequence

UniProt ID	Protein name	Organism	Protein length	Signal peptide position	PDB structure
Q9VQ62	NPC2_DROME	*Drosophila melanogaster* (fruit fly)	148	19	1NEP
P61916	NPC2_HUMAN	*Homo sapiens* (human)	151	20	1NEP
Q9Z0J0	NPC2_MOUSE	*Mus musculus* (mouse)	149	20	1NEP
P79345	NPC2_BOVIN	*Bos taurus* (domestic cattle)	149	20	2HKA
O97763	NPC2_PIG	*Sus scrofa* (pig)	149	20	1NEP
P61917	NPC2_PANTR	*Pan troglodytes* (chimpanzee)	151	20	1NEP
P61918	NPC2_MACFA	*Macaca fascicularis* (crab-eating macaque)	151	20	1NEP
Q28895	NPC2_CANLF	*Canis lupus familiaris* (dog)	149	22	1NEP
Q9DGJ3	NPC2_DANRE	*Danio rerio* (zebrafish)	149	20	1NEP

### Curation of sequences

All inferred *C. sinensis* NPC2-like proteins were individually curated in three steps (Fig. 1). First, gene regions (including exonic and intronic nucleotide sequences) encoding *C. sinensis* NPC2-like proteins were extracted from each of the two draft genomes (representing Chinese and Korean isolates) *via* the corresponding “general feature format” (GFF) file employing the gffread tool v.0.11.4 [35]. A reciprocal BLASTn v.2.2.29 search against the *C. sinensis* NPC2-like gene sets (for both isolates) was then conducted to infer gene orthology. Second, *C. sinensis* NPC2-like proteins from each isolate (Chinese or Korean) were reciprocally mapped to the genome of the alternative isolate using BLAT v.34x12 [36], and a new gene model was inferred based on this mapping employing the program Exonerate v.2.2.0 [37].

### Analysis of transcription

Available RNA-Seq data (testis, sucker, muscle, ovary, adult, 8-week adult, metacercaria, 2-week juvenile; ENA/SRA accession numbers: ERR604978–ERR604981, SRR189060, SRR6188894–SRR6188896) for *C. sinensis* were mapped to each reference genome using HISAT2 [38]. From these mapped data (stored in the BAM format), read counts were inferred using the corresponding GFF files and employing FeatureCounts v.1.6.4 [39]. Read counts from each library were then normalised to counts per million (CPM) using the *edgeR* package v.3.26.8 [40]. A heatmap matrix of CPM per gene per library was created using the *ggtree* R package v.1.16.6 [34].

### Protein annotation

Signal peptides and their cleavage sites were inferred for *C. sinensis* NPC2-like proteins using the SignalP-5.0 [41]. The structures of the mature *C. sinensis* NPC2-like proteins (i.e. without their predicted signal peptide domain) were modelled and annotated using the program I-TASSER v.4.4 [42] and compared with the crystal structures of NPC2s from *Bos taurus* (cow; Protein Data Bank (PDB) accession 2HKA chains A and C) [19] and *Camponotus japonicus* (Japanese carpenter ant; PDB accession 3WEA chain A) [43] employing the program UCSF Chimera v.1.9 [44].

### Phylogenetic analysis

The mRNA sequences encoding *C. sinensis* NPC2-like proteins were extracted from each assembled genome sequence *via* their corresponding GFF files employing gffread. Codons of *C. sinensis* NPC2-like sequences were aligned using a translated protein sequence alignment employing MACSE v.2.03 [45]. Gaps were removed from the nucleotide alignment using trimAl v.1.4.rev15 [46] using the -gappyout option. The Akaike Information Criteria (AIC) test in ModelFinder [47] selected the general time reversible model of evolution for subsequent phylogenetic analyses. Bayesian phylogenetic inference (BI) was determined using Markov chain Monte Carlo (MCMC) analysis in MrBayes [48]. Two million generations of MCMC analysis were performed, and trees were recorded every 200th generation. At this point, the standard deviation of split frequencies was < 0.01, and the potential scale reduction factor (PSRF) approached 1. Consensus trees (50% majority rule) were generated using the final 75% of trees. Trees were annotated and enhanced using the *ggtree* R package v.1.16.6 [34], and nodal support values on trees were indicated as posterior probabilities (pp).

## Results

### NPC2-like proteins of *Clonorchis sinensis*

Based on predicted protein sequence homology, 72 of 14,538 Korean *C. sinensis* proteins and 71 of 13,634 Chinese *C. sinensis* proteins were homologous to one or more NPC2 proteins in SWISS-PROT (Fig. 1, Additional file 1: Table S1). Sequence homology between NPC2 proteins and their best matched *C. sinensis* proteins was mostly low (20.0–47.6 % amino acid identity), and sequence alignment coverage was highly variable (14–100%). In total, 55 Korean and 56 Chinese NPC2 homologues were reciprocal BLASTp matches (E-value cut-off: 10) to NCBI-nr proteins annotated as NPC2-like or those without a protein description and were thus retained (Fig. 1, Additional file 1: Table S1). Of those, 35 Korean and 27 Chinese *C. sinensis* proteins contained at least one ML domain, with an average conserved domain length of 107 amino acids (Fig. 1, Additional file 1: Table S1). Two conserved ML domains were predicted in three Korean *C. sinensis* protein sequences (Cs-k2.gene14549, Cs-k2.gene14290 and Cs-k2.gene14112). Based on protein sequence homology to NPC2 and the presence of a conserved ML domain (Fig. 1), 35 and 27 NPC2-like proteins were predicted to be encoded in the Korean and Chinese *C. sinensis* gene sets, respectively. One Chinese protein sequence (csin112467) that shared no significant sequence homology to NPC2 proteins, but contained a conserved ML domain, was also retained for subsequent curation (Fig. 1).

### Gene models

Reciprocal nucleotide alignments of Korean and Chinese *C. sinensis* NPC2-like gene regions (including introns) and mapping to the alternative genome assemblies identified paired orthologues between the two isolates. Nine pairs of NPC2-like gene models were consistent in gene model structure and overall sequence length. Based on reciprocal nucleotide matches across exonic and intronic regions, we identified 11 additional orthologous pairs with unresolved variation in the first exon positions and lengths. Three of the Korean *C. sinensis* genes identified by reciprocal nucleotide alignments (i.e. Cs-k2.gene992, Cs-k2.gene8673 and Cs-k2.gene14547) were removed as they did not encode a conserved ML domain or share amino acid sequence homology with NPC2 proteins from SWISS-PROT. In contrast, four Chinese *C. sinensis* genes (csin101111, csin103126, csin111538 and csin111895) were added based on mapping of Korean NPC2-like gene sequences to the genomic region encoding these genes. Six Korean gene models, although aligning to the Chinese genome, only aligned partially to the corresponding Chinese gene model in this genomic region, and available sequence data were insufficient to resolve a single gene model for both isolates. Eight Korean *C. sinensis* genes were not identified in the Chinese genome, and four Chinese *C. sinensis* genes were not identified in the Korean genome. Taken together, 35 Korean and 32 Chinese NPC2-like protein genes were retained.

### Tertiary structures and functional annotation

The tertiary structures of 35 Korean and 32 Chinese *C. sinensis* NPC2-like proteins were modelled using I-TASSER (Additional file 1: Table S2). The I-TASSER model confidence (C-) scores ranged from -5 (lowest confidence) to 1.29 (highest confidence). For comparison, I-TASSER models were also inferred for nine curated NPC2 proteins from SWISS-PROT (Table 1). For these proteins, the model C-scores ranged from 1.30 to 1.46. Eleven Korean and nine Chinese *C. sinensis* NPC2-like proteins had predicted structures with C-scores of ≥ 1. These high-confidence models were retained for further analysis. Two Korean and three Chinese NPC2-like proteins had C-scores of < -4 and were thus low-confidence predictions. Proteins Cs-k2.gene6404 (Korean isolate) and csin102672 (Chinese isolate) had the highest paired ortholog C-scores (1.26). Chinese NPC2-like proteins csin107773 and csin111438 had the highest (1.29) and lowest C-score (-5), respectively. Of note, six Korean and Chinese NCP2-like proteins with a C-score of < -4 were not predicted to retain a signal peptide region (Additional file 1: Table S2). Based on protein structurally close to the target in PDB, 2HKA chain A (NPC2 from cow) and 3WEA chain A (NPC2 from the Japanese carpenter ant) had the highest and second-highest structural similarity to *C. sinensis* NPC2-like models (representing 44 and 5 of all structures predicted, respectively; Additional file 1: Table S2). The most commonly predicted ligand (73%) was cholesterol (C3S).

### Phylogenetic relationships

**Fig. 2:**
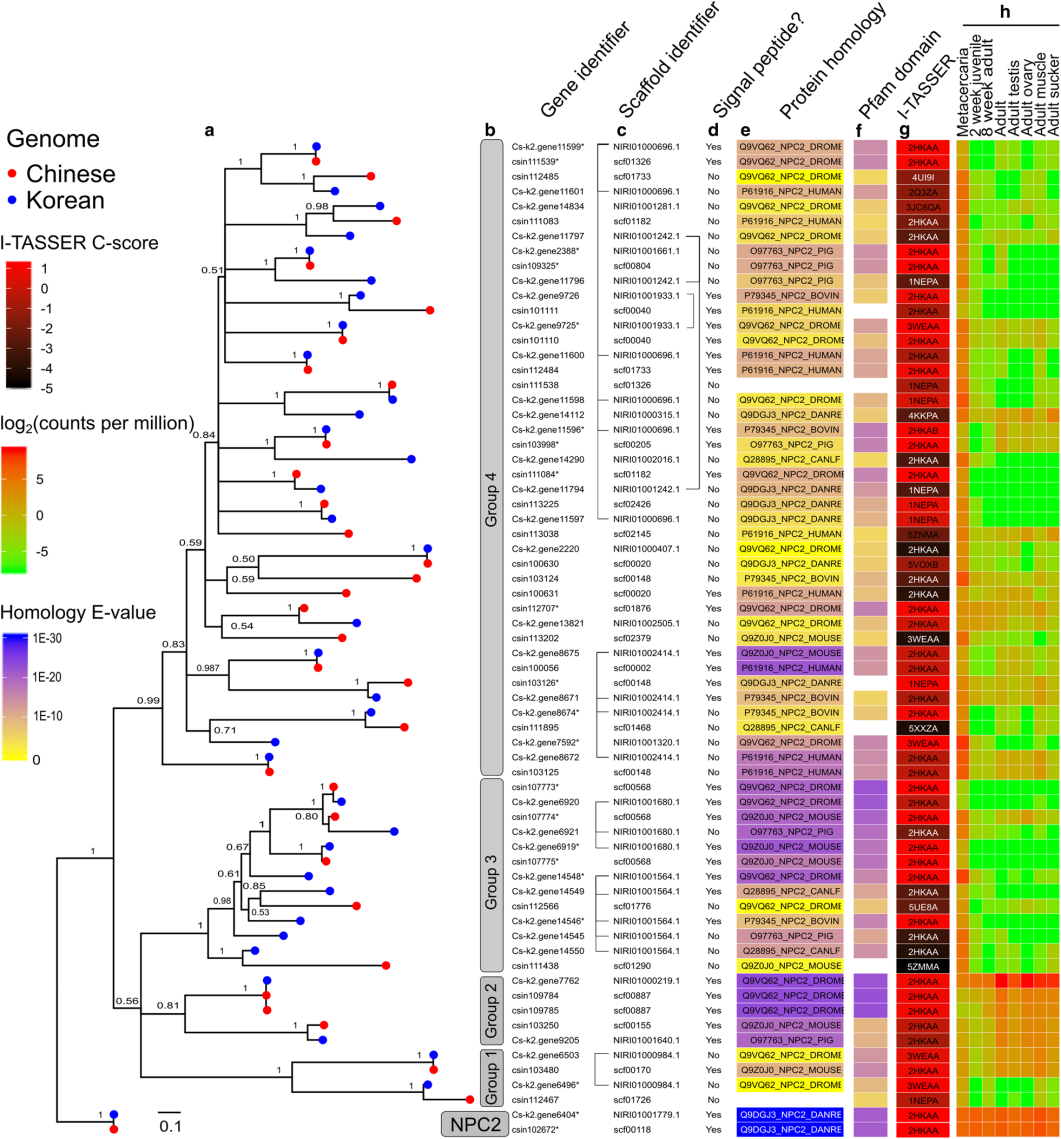
Phylogenetic relationships among curated NPC2 proteins identified in two genomes of *Clonorchis sinensis* (**a**). Curated SWISS-PROT NPC2 proteins and known PDB structures for NPC2-like proteins were used as an outgroup. For each *C. sinensis* gene, their predicted Chinese (red) and Korean (blue) orthologues are presented as pairs in the phylogenetic tree. For each gene, the phylogenetic grouping (**b**) and the encoding scaffold (**c**) are listed. For all encoded protein sequences, the presence of a signal peptide (**d**), homology to canonical SWISS-PROT NPC2 proteins (**e**) and the presence of a conserved Pfam ML domain (PF02221) (**f**) are indicated. For representative pairs of orthologous proteins, the predicted quality of the I-TASSER model (C-score; “confidence score”) and the best matched PDB structure is shown (**g**). Transcription levels for each NPC2-like protein-encoding gene in available developmental and tissue-specific RNA-Seq libraries are included as a heatmap (**h**)

The phylogenetic relationship among *C. sinensis* NPC2-like proteins was determined using aligned coding domains; the resulting tree (Fig. 2a) was annotated with experimental data (Fig. 2b-h). NPC2-like proteins of *C. sinensis* clustered in four well-supported groups (pp = 0.81–1.0) that contained 27 paired orthologues, with eight and four proteins being unique to the Korean and Chinese isolates, respectively (Fig. 2b). The Korean and Chinese *C. sinensis* proteins most similar to NPC2 proteins from SWISS-PROT clustered within group 2 (E-value 1.23E^−20^–2.91E^−24^), whereas *C. sinensis* sequences that were least similar to NPC2 proteins from SWISS-PROT were within group 1 (E-value 2.64E^−10^–4.65) or group 4 (E-value 6.12E^−23^–5.28). Interestingly, NPC2-like proteins were frequently encoded on the same genome scaffold (Fig. 2c). For example, four groups of two to six Korean NPC2-like proteins within group 4 were encoded on the same scaffold, whereas one and two groups of Korean proteins (*n *= 2–5) in group 3 and group 1, respectively, were encoded on the same scaffold.

Based on available *C. sinensis* transcriptomic sequence data, NPC2-like protein-encoding genes within group 2 showed evidence of transcription in all developmental stages (metacercaria, juvenile and adult) and adult tissues (testis, ovary, muscle and sucker) (Fig. 2h). Most NPC2-like protein-encoding genes in *C. sinensis* showed moderate to high transcription in the metacercarial stage. Genes in group 1 showed the lowest (overall) transcription levels across all stages and tissues. The gene Cs-k2.gene7762 (Korean isolate) showed the highest transcription overall, and was highly transcribed in all stages and tissues studied.

Employing curated data sets (Fig. 2 and Additional file 1: Table S2), the paired orthologues Cs-k2.gene6404 and csin102672 were inferred to encode canonical NPC2 proteins. Transcriptomic evidence supported their constitutive transcription in all stages and in adult tissues. In addition, the proteins encoded by these genes were the most similar to curated NPC2 proteins from SWISS-PROT, and their predicted tertiary structures had the highest C-scores.

### Evidence for structural conservation

**Fig. 3:**
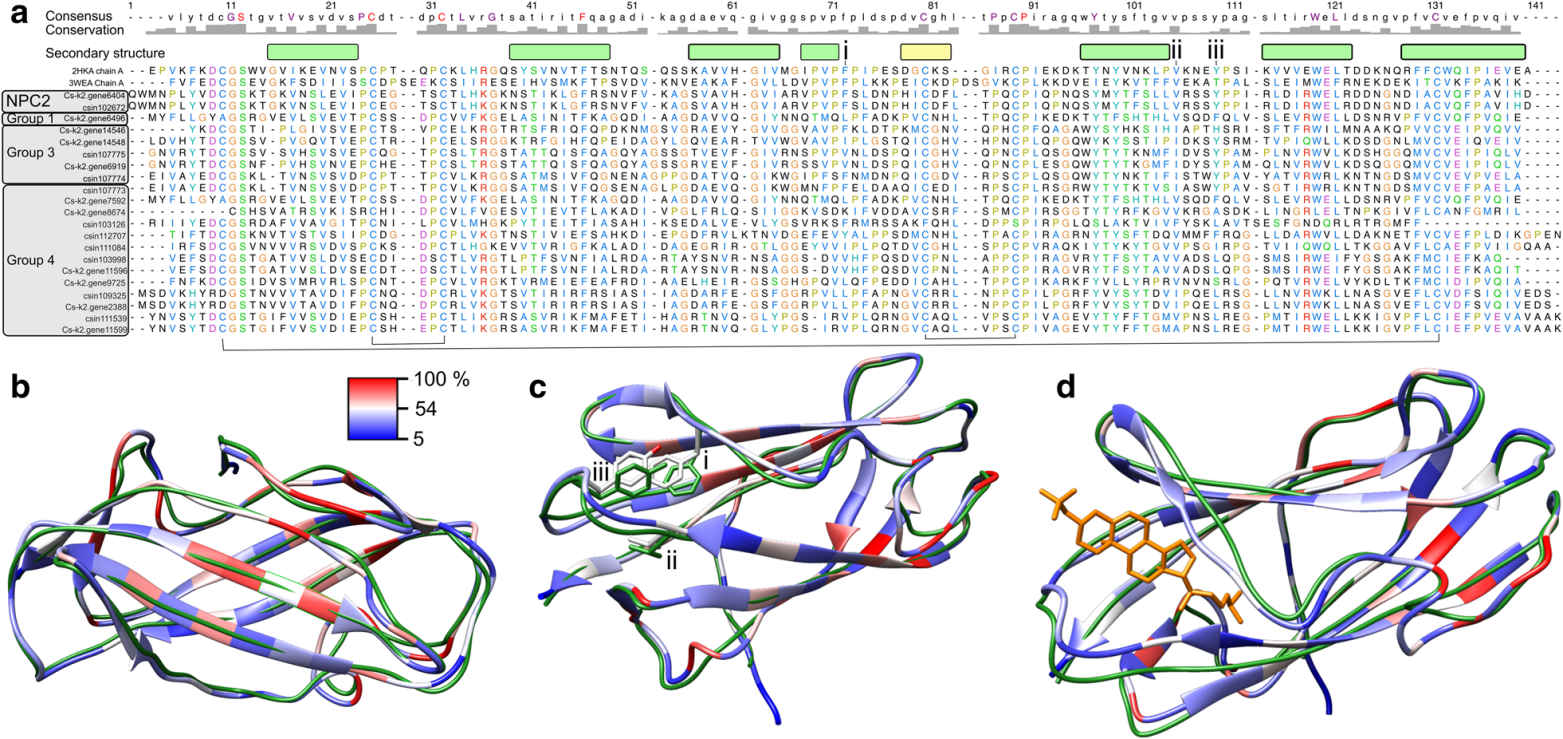
Assessment of structural conservation in *Clonorchis sinensis* NPC2 and NPC2-like proteins. Predicted structures of 21 *C. sinensis* NPC2 and NPC2-like proteins were aligned to assess conservation relative to two reference NPC2 protein structures (2HKA and 3WEA). **a** Alignment of NPC2 and NPC2-like sequences and conserved barrel with seven-stranded β-sandwich folds (shown in green) fixed by three disulfide bonds (Cys-8-Cys-121, Cys-23-Cys-28, and Cys-74-Cys-80; indicated by black lines below the alignment). **b** Positioning of loops in *C. sinensis* NPC2 models was modelled on a closed sterol binding pocket. Conserved residues are shown in red, variable residues are shown in blue and fewer variable residues are shown in white. **c** Hydrophobic tunnel from an opening created by three β-sandwich loops and highlighting three amino acid residues important for binding cholesterol (Val-105, **i**; Tyr-109, **ii**; and Phe-73, **iii**). **d** A fully open pocket in the presence of bound cholesterol sulphate (orange)

Predicted high-confidence (C-score: ≥ 1) structures of 21 *C. sinensis* NPC2 and NPC2-like proteins (11 and 10 for Korean and Chinese isolates, respectively) were aligned with the two most similar PDB reference structures (2HKA and 3WEA) to assess conservation (Fig. 3). Most proteins were predicted to retain a conserved Ig-like β-sandwich fold conformation with seven-stranded β-sandwich folds fixed by three disulfide bonds (Cys-8-Cys-121, Cys-23-Cys-28, and Cys-74-Cys-80) and a large cavity in the interior of a protein barrel (Fig. 3a–c). In mammals, NPC2 binds cholesterol in the deep hydrophobic tunnel created by the βa and βb-βc loops (Fig. 3c) [19]. Predicted *C. sinensis* NPC2 and NPC2-like models were more similar to the resolved structure in the absence of bound cholesterol sulphate (2HKA chain A; Fig. 3b, c) than the resolved structure with an open pocket in the presence of bound cholesterol sulphate (2HKA chain C; Fig. 3d). Importantly, only Cs-k2.gene6404 and csin102672 proteins retained the three amino acid residues (Val-105, Tyr-109 and Phe-73) that are required for cholesterol binding [19] (Fig. 3a, c), further supporting their annotation as canonical NPC2 proteins.

## Discussion

This study conducted comparative genomic analyses to explore the substantial expansion of a NPC2-like protein family in *C. sinensis*. Using a bioinformatic workflow, we curated 35 and 32 *C. sinensis* NPC2-like proteins representing the Korean and Chinese isolates, respectively, clustered them into four distinct phylogenetic groups, predicted their tertiary structures and recorded transcriptional levels for the genes encoding these proteins in distinct developmental stages and tissues. These data provide evidence for the presence of a structurally conserved canonical form of NPC2 in *C. sinensis* and were used to explore the functional implications of genetic variation among members of the *C. sinensis* NPC2-like protein family.

Earlier characterisations of human NPC2 homologues [20] in liver flukes revealed an expansion of this protein family [16–18]. The present study predicted a higher number of NPC2-like proteins in *C. sinensis* compared with earlier studies and provides evidence of relatively high levels of genetic conservation between the Korean and Chinese isolates. To date, investigations of other platyhelminths or most other eukaryotes have reported only one or two copies of NPC2-like proteins [20], which likely retain a conserved role in sterol transport from the late endosome and/or lysosomes, together with conserved NPC1 proteins (*C. sinensis* gene identifiers Cs-k2.gene5262 and csin107525) [49]. The genetic mechanisms in *C. sinensis* that have led to a relatively rapid expansion and genetic diversification beyond a single, canonical NPC2-like gene remain to be investigated in detail. Clustering of *C. sinensis* NCP2-like proteins on the same draft genomic scaffolds (for each isolate) suggests gene duplication mechanisms are likely to be involved, including unequal crossing-over [50], retrotransposition [51], duplicated DNA transposition [52] and/or polyploidisation [53]. In arthropods, gene duplication events appear to have led to a similar expansion of ML domain-containing proteins (which includes NPC2-like proteins). For example, MD-2 or NPC2*-*like protein family expansions in insects have been recorded in *Anopheles gambiae* (13 copies), *Aedes aegypti* (15 copies), *Tribolium castaneum* (8 copies) and *Drosophila melanogaster* (8 copies) [20, 21]. The extent of NPC2 gene duplication events in other flatworms remains to be determined. Further characterisation of NPC2-like proteins encoded in all available genomes of all members of the phylum Platyhelminthes should provide useful insights into the evolution of this family of proteins as well as their functional roles in free-living and parasitic taxa.

Molecular characterisations of several arthropod ML-proteins support neofunctionalisation arising from gene duplication, with diversified ML proteins playing crucial roles in steroid biosynthesis [54], immunity [55] and chemoreception [23–26]. For example, arthropod ML proteins can act as receptors (e.g. “pattern recognition receptors”) or co-receptors for various ligands to modulate innate immune signalling pathways [21]. In addition, several NPC2-like proteins are highly expressed in the chemosensory organs of ants [43], ticks [24] and spiders [56], where they are reported to play a key role in chemoreception, by acting as carriers of semiochemicals [23–25]. As *C. sinensis* is taxonomically and evolutionarily distinct from arthropods, the gene expansion events in arthropods and liver flukes appear to be independent as they do not share common ancestry. Therefore, it is unlikely that the function of *C. sinensis* NPC2-like proteins can be inferred from amino acid sequence homology. However, the predominant transcription of most NPC2-like *C. sinensis* proteins in the metacercarial stage does provide support for a role in chemoreception; the infective metacercarial stage is exposed to a hostile environment which requires molecular mechanisms facilitating survival, adaptation, migration and development [57], and there is increasing evidence that bile stimulates the expression of particular genes involved in these processes [58]. Importantly, chemoreception has been shown to play a criticial role in the ability of newly excysted juveniles to locate the ampulla of Vater and migrate into the biliary system [15, 59]. The abundance of transcripts of most NPC2-like protein genes in the metacercarial stage might be indicative of an important role for these proteins during excystation, initial growth and development and/or migration. Whether these NPC2-like proteins are expressed and/or linked to the chemotactic behaviour of *C. sinensis* warrants detailed investigation. In addition to roles in chemotaxis, a high level of transcription of some of the NPC2-encoding genes in adult tissues (including reproductive tissues and sucker) might suggest broader roles in lipid metabolism, feeding and/or reproduction [16, 20]. In other parasites, including intracellular protists [60, 61] and schistosomes [62], which cannot synthesise cholesterol, these molecules may have functions in nutrient uptake, immune evasion and/or energy storage. If *C. sinensis* is unable to synthesise cholesterol, as is the case for the related liver fluke *O. viverrini* [16], canonical NPC2 in *C. sinensis* might assume similar functions.

In this study, our established bioinformatics workflow [63] assisted in the prediction of a conserved set (family) of *C. sinensis* NPC2-like proteins. Evidence that two pairs of NPC2-like protein orthologues (Cs-k2.gene11598/csin11538 and Cs-k2.gene9726/csin101111) undergo positive selection [17] lends support for a recent expansion of this protein family. Interestingly, a small number of gene encoding these proteins (*n* = 4–8) were present exclusively in the Korean or Chinese isolate; whether these genes/proteins have evolved recently and are indeed isolate-specific remains to be established. Resolving the final copy numbers of NPC2-like protein genes in *C. sinensis* will necessitate the sequencing and assembly of complete, chromosome-contiguous genomes using ‘third-generation’ technologies [64]. This would also pave the way to detailed comparative genome analyses.

## Conclusions

In the present study, we used comparative genomics and functional annotation tools to explore a novel family of NPC2-like proteins in *C. sinensis*. Based on our findings, we propose that these proteins are involved in binding and transportation of sterols and/or other lipids throughout the life-cycle of this parasite. The curated set of these proteins presented herein provides a solid foundation for future investigations of the molecular functions of NPC2-like proteins in *C. sinensis* and other liver flukes.

## Supplementary information

**Additional file 1: Table S1.** *Clonorchis sinensis* NPC2-like proteins (Korean and Chinese isolates) with homology to SWISS-PROT NPC2 proteins, NPC2-like proteins in the NCBI-nr database and/or conserved ML domain (PF02221). **Table S2.** Predicted functional annotation of 35 Korean and 32 Chinese *Clonorchis sinensis* NPC2-like proteins and nine SWISS-PROT NPC2 proteins using SignalP, SWISS-PROT, InterProScan and I-TASSER. For each NPC2-like protein, the best I-TASSER model is shown, including the associated C-score, estimated topology modelling (TM) score, root-mean-square deviation of atomic positions (RMSD), predicted function, as well as the most similar target in the PDB database. Predicted transcription in different developmental stages and adult tissues is shown as log2 counts per million.

## Data Availability

All data generated or analysed during this study are included in this published article and its additional file.

## Abbreviations

AIC: Akaike information criteria
BI: Bayesian inference
BLAST: basic local alignment tool
BLASTn: nucleotide BLAST
BLASTp: protein BLAST
BLAT: BLAST-like alignment tool
C-score: I-TASSER model confidence score
CCA: cholangiocarcinoma
CPM: counts per million
ENA: European Nucleotide Archive
GFF: general feature format
I-TASSER: iterative threading assembly refinement
IARC: International Agency for Research on Cancer
LBPs: lipid-binding proteins
MCMC: Markov chain Monte Carlo
MD-2: myeloid differentiation factor-2
ML: MD-2-related lipid-binding domain
NCBI-nr: National Center for Biotechnology Information nonredundant database
NPC2: Niemann-pick type C2
NTD: neglected tropical disease
PDB: protein data bank
pp: posterior probability
PSRF: potential scale reduction factor
RMSD: root-mean-square deviation of atomic positions
RNA-Seq: RNA sequencing
SRA: sequence read archive
TM-score: I-TASSER topology modelling score
